# Preseason and In-Season High-Speed Running Demands of 2 Professional Australian Rules Football Teams

**DOI:** 10.1177/19417381241265114

**Published:** 2024-08-22

**Authors:** Brock W. Freeman, Scott W. Talpey, Lachlan P. James, Russell J. Rayner, Warren B. Young

**Affiliations:** † Institute of Health and Wellbeing, Federation University Australia, Ballarat, Australia; ‡ School of Health Sciences and Physiotherapy, The University of Notre Dame Australia, Fremantle, Australia; § School of Allied Health, Human Services, and Sport, La Trobe University, Melbourne, Australia; ‖ School of Allied Health, Exercise and Sports Sciences, Charles Sturt University, Port Macquarie, Australia

**Keywords:** field-sports, global positioning system, load management, sprinting, training and monitoring

## Abstract

**Background:**

Australian Rules Football athletes complete long preseasons, yet injuries occur frequently at early stages of the competitive season. Little is known about the high-speed running (HSR) prescription during a preseason or whether players are adequately prepared for competition. This study described absolute and relative preseason and in-season HSR demands of 2 professional Australian football teams.

**Hypothesis:**

HSR and sprinting volumes are significantly lower in elite Australian Rules football athletes during in-season compared with preseason.

**Study Design:**

Cohort study.

**Level of Evidence:**

Level 3.

**Methods:**

During the 2019 season, HSR volume was collected for 2 professional Australian football teams (n = 55). Individual maximum speeds (V_max_) were captured to calculate relative running speed thresholds, as reported in 5% increments from 70%V_max_ to 100%V_max_.

**Results:**

Weekly volume of running above 70%V_max_ (*P* = 0.01; *r* = 0.56) and 80%V_max_ (*P* = 0.01; *r* = 0.58) was significantly greater in the preseason than the in-season. The weekly volume completed above 90%V_max_ was not significantly greater in the preseason than the in-season (*P* = 0.10; *r* = 0.22). Individual variation in the distance completed at specific percentages of V_max_ expressed as a coefficient of variation was reported as 51% at 71% to 80%V_max_, 39% at 81% to 90%V_max_, and 41% at 91% to 100%V_max_.

**Conclusion:**

The volume of HSR completed by athletes is far greater in the initial 4 weeks of the preseason than in any other point in preseason or competitive phases. At the individual level, there is substantial variation in the distance covered. This supports the concept of a heavily individualized approach to high-speed prescription and monitoring.

**Clinical Relevance:**

Practitioners should carefully consider individual variation regarding sprinting volume during both preseason and in-season when prescribing and monitoring training to improve on-field performance and reduce the risk of injury.

There is considerable scientific, practical, and public interest in the training and competition demands of Australian Rules Football (AF).^11^ Researchers, alongside practitioners, have sought to quantify and understand the demands of AF to better prepare athletes for competition. This is a result of league-wide use of global positioning systems (GPS) to collect data that are used primarily for performance and injury prevention.^20^ The match demands of professional AF athletes have been previously established in peer-reviewed literature,^6,7,10,11,13,36,40,50^ highlighting the large amounts of high-speed running (HSR) and sprinting completed by athletes. As a byproduct of these advancements, the public has a growing interest in the performance metrics of their favorite athletes. For example, the Australian Football League (AFL) provides real-time, in-game measures of individual athlete running volume and intensity to fans via their mobile application. This availability of information signals the need for a better understanding of the data provided to the masses.

HSR is a commonly used umbrella term in practice and peer-reviewed literature. Typically, an athlete is performing HSR when they exceed a certain absolute or relative running speed threshold.^26^ A recent AFL injury report highlights that lower-limb soft tissue injuries occur more frequently during match-play—a finding consistent with other literature.^1,37^ Data collected from the online injury report of the AFL (Appendix Figure A1, available in the online version of this article) highlight that the initial 8 weeks of the 2018 season displayed a higher weekly injury rate than both the 2018 weekly average and the 2017 weekly average. Therefore, given the noted risk of injury with HSR, particularly in the early in-season phase, it is reasonable to question whether preseason demands are adequately preparing athletes for the demands of competition.

Specifically, in professional AF, there is an extensive preseason that aims to build the physical capabilities of the athletes so that they can perform optimally during the competitive phase of the season and reduce the risk of injury. It is logical that HSR and sprinting should serve as key foci in a preseason program due to the previously reported high in-season demands.^6,7,10,11,13,36,40,50^ Typically, athletes who complete higher volumes of training in the preseason are able to cover more distance during in-season match activities.^47^ Moreover, a higher session rating of perceived exertion in the late preseason has the biggest influence on performance during the first 4 matches of the season.^32^ However, the relationship between preseason and in-season HSR load is unknown.

Previous investigations into muscle kinematics and kinetics have reported large increases in the mechanical demands of the lower-limb muscles as running speed increases.^8,16,38,43^ A prominent example is the hamstring muscles, where increases are amplified as running speed exceeds 80%V_max_, with significant changes observed across 5% increments up until 100% of maximum speed.^8^ This provides the rationale that exposing players to speeds closer to 100%V_max_ is important as these speeds place greater demands on the lower-leg muscles and likely help build soft-tissue resilience at higher speeds.^20^ The relative high demands required during sprinting provide a rationale for prescribing training to improve sprint performance in small doses. Small doses of exposure to maximal speed sprinting may provide both a protective and performance enhancing effect for the athlete.^20^ An example of this may be achieving >95% of maximum running speed (V_max_) on 2 to 3 occasions during a microcycle (ie, 7-10 days). This example would allow for a consistent chronic training load to remain throughout the season.^17,22,37^

Multiple findings have implicated the importance of consistent exposure to HSR and sprinting to mediate injury risk.^17,30,37^ However, if HSR and sprinting loads for field sport athletes are evaluated in groups, some athletes might be exposed to very little HSR, whereas some athletes may potentially be overexposed. Therefore, establishing the individual variation would provide practitioners with effective information that could be used when planning training cycles. To date, there is no evidence evaluating the differences in HSR and sprinting between the preseason and the in-season phases of professional AF. Due to the broad definitions of HSR, there is no published evidence outlining the differences between smaller, more specific speed thresholds that comprise HSR and sprinting. The primary hypothesis for this study was that the athletes would complete significantly more HSR and sprinting volume during the preseason compared with in-season. It was further hypothesized that some athletes would not achieve maximum speed (>95%V_max_) during the in-season period. Therefore, the purpose of this study was to determine the amount of HSR completed by elite AF athletes from 2 clubs during the preseason and compare this with the in-season values. The secondary aim was to investigate individual variances from the mean total distances in each speed threshold.

## Methods

Following the 2019 AFL season, GPS files were obtained from 2 professional AF clubs. An initial sample of 90 male athletes was included in the study. For data to be included in the investigation, the athletes were required to have completed a minimum of 80% of the clubs planned training sessions and compete in at least 3 of the first 8 rounds of the 2019 season. This reduced the initial sample to 55 athletes (see Table 1 for athlete characteristics).

**Table 1.** Participant characteristics (mean ± SD)

	Team A	Team B	Pooled Mean
Age, years	24 ± 3.9	24 ± 3.3	24 ± 3.6
Height, m	1.87 ± 0.08	1.88 ± 0.07	1.87 ± 0.80
Body mass, kg	85 ± 8.5	86 ± 8.8	86 ± 9.0
Sessions completed, preseason	40 ± 4.1	28 ± 3.2	34 ± 3.6
Sessions completed, in-season	31 ± 3.1	23 ± 2.3	27 ± 2.7

An observational cohort study design was employed to determine the volume of HSR performed at arbitrary predetermined thresholds set by the manufacturer (Catapult Sports) and individualized intensities during the preseason and early competition phases of the 2019 AFL season. De-identified GPS files (n = 4174) from both training sessions and competition from the 2019 AFL season were obtained from 2 participating AFL Clubs. This project received ethical approval from the University Human Research Ethics Committee (Approval No., C20-003).

The first preseason session commenced when the athletes returned from their Christmas break on January 14, 2019, and concluded after the final preseason practice match on March 17, 2019. The in-season period began on March 18 and included the first 8 rounds of the season. The rationale for limiting the in-season period of data collection to the first 8 rounds was due to the established link in preseason training load to early season injury risk.^47^ Athletes were fitted with Catapult S5 GPS units (Catapult Sports) sampling at 10 Hz and were worn for all field sessions (training and matches) during the data collection period. Previous research has demonstrated these units to be reliable (intraclass correlation coefficient, 0.89) to collect HSR distance.^27^ The GPS units were attached to the body using either a specially designed spandex GPS bib or placed in the athlete’s jersey. This ensures the unit is centered securely between the scapulae on the upper back.

Once GPS files were imported into the processing software (Catapult OpenField Version 1.21.1, Catapult Sports), each individual file was analyzed and cleaned, and any abnormal data were removed; this can occur when there has been a loss of satellite connection (most likely the unit is still on and an athlete has walked indoors), which can sometimes result in erroneous spikes in data as the unit attempts to regain satellite connection. To determine distance covered in each speed band, athletes were required to spend >1 second at a running speed above the minimum of the respective threshold. Total distance (meters) was calculated as the sum of all absolute velocity bands for the given session. Therefore, to establish the weekly totals, the total distance from each session within the week (Monday through Sunday) was summed and then divided by the number of sessions in the respective week. This process was repeated for all absolute and relative speed bands.

The absolute speed bands selected for this study were adapted from a series of studies investigating the match demands of different field sports (Table 2). Therefore, the following speed bands were selected for this study to equally represent previously used speed thresholds: <5.5 m s^-1^, 5.51 to 7 m s^-1^, 7.01 to 8.5 m s^-1^, 8.51 to 10 m s^-1^, and >10 m s^-1^.

**Table 2.** Speed bands used to quantify the running demands of AF and other male field sports

Author	Sport	Jogging (m s^-1^)	Running (m s^-1^)	Striding (m s^-1^)	Sprinting (m s^-1^)
Aughey et al^2^	AF	4.2	5	-	7
Bauer et al^5^	AF	2.5	3.5	5.5	7
Brewer et al^6^	AF	-	4.2	-	5.5
Burgess et al^7^	AF	-	-	-	5.5
Colby et al^10^	AF	-	3.5	-	-
Coutts et al^11^	AF	1.9	4	5.5	6.4
Duhig et al^17^	AF	-	-	6.6	-
Ruddy et al^37^	AF	-	-	6.6	-
Stares et al^40^	AF	-	-	-	-
Sullivan et al^41^	AF	-	4	-	-
Wisbey et al^50^	AF	-	3.4	-	5
Mean ± SD		2.9 ± 1.2	3.9 ± 0.6	6.1 ± 0.6	6.1 ± 0.9
Dwyer et al^18^	AF + Other	2.1	3.6	-	5.6
Varley et al^46^	AF + Other	-	-	5.5	7
Austin et al^3^	Other	3.3	3.9	5.5	6.7
Bangsbo et al^4^	Other	1.9	2.8	-	6.7
Clarke et al^9^	Other	-	3.5	-	-
Curtis et al^12^	Other	2	4	-	6
Di Salvo et al^14^	Other	-	3	-	6.4
Dogramaci et al^15^	Other	2	4	-	6
Impellizzeri et al^23^	Other	2.1	3.3	-	6
Jennings et al^25^	Other	-	4.2	-	-
Krustrup et al^29^	Other	1.9	2.8	-	6
Malone et al^30^	Other	-	4	-	5.5
McClellan et al^33^	Other	1.6	2.8	3.8	5.6
Mohr et al^34^	Other	1.9	2.8	-	6.7
Quarrie et al^35^	Other	2	4	6	8
Sirotic et al^39^	Other	2	3.6	5.2	6.7
Tee et al^42^	Other	2	4	-	6
Twist et al^45^	Other	-	3.6	-	5
Wehbe et al^49^	Other	3.3	4.4	5	7
Yamamoto et al^51^	Other	2	4	5.5	6.9
Mean ± SD		2.2 ± 0.5	3.6 ± 0.5	5.2 ± 0.7	6.3 ± 0.7

AF, Australian Rules Football; Other, other sports.

Initially, each athlete’s maximal speed was determined by the greatest maximal speed attained from the GPS unit during the first 4 weeks of preseason training. If the athlete recorded a higher maximum speed (V_max_) in the following weeks of analysis, this value was updated, and relative speed zones were recalculated. Relative speed bands were established with a 5% change between each band: 70% to 75%V_max_, 76% to 80%V_max_, 81% to 85%V_max_, 86% to 90%V_max_, 91% to 95%V_max_, and 96% to 100%V_max_. Further analyses were then completed with a 10% increase in each speed band: 70%V_max_, 80%V_max_, and 90%V_max_, to provide a broader picture of the demands with speed bands that might be considered more practical in an applied setting. This approach was used because recent research has indicated that AF high performance managers do not conduct dedicated speed testing and therefore attaining maximal speed capabilities of athletes via GPS variables is current industry practice.^2^

Participant data from Club A and Club B were pooled to investigate general trends, whereas individual club data allowed for further analysis and comparison between clubs. Python Version 3.10 was used for data analysis, using Pandas and NumPy for data handling, as well as SciPy and statsmodels for statistical tests. Descriptive statistics (means and standard deviations) were calculated to give an overview of the variability in distance covered in absolute and relative speed bands by athletes across both the preseason and in-season. Normality and homoscedasticity were assessed using Shapiro-Wilk and Levene’s tests, which revealed violations of the assumptions required for parametric tests. As such, Wilcoxon signed-rank tests were used to compare the running volume between the preseason and in-season periods for pooled data, Club A, and Club B. This process was also completed to compare differences between Club A and Club B. *P* values were corrected for multiple comparisons using the Benjamini-Hochberg procedure to control the false discovery rate. Effect sizes were calculated using the formula^44^:

$$r=\frac{\left|Z\right|}{\sqrt{n}}$$

Individual variability for distance completed in absolute and relative speed bands was compared with the group average for both the preseason and in-season and then presented graphically.

## Results

**Figure 1.**
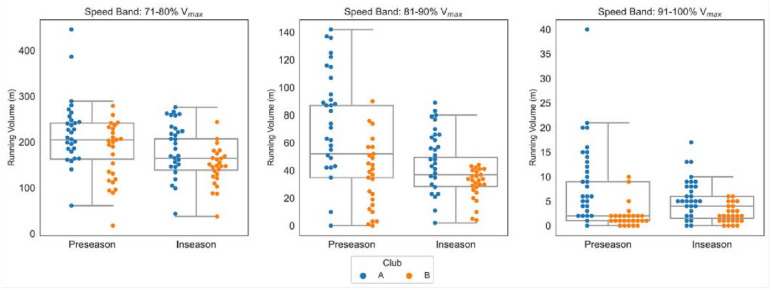
Comparisons of 71% to 80%V_max_, 81% to 90%V_max_, and 91% to 100%V_max_ running volumes completed in the preseason and in-season for both Club A and Club B. Median and interquartile range indicate pooled data from both clubs, and individual datapoints are denoted by the legend. V_max_, maximum speed.

Analyses revealed significantly greater distance was covered in preseason when compared with in-season running in the 71% to 80%V_max_ speed band (*P* < 0.01, *r* = 0.56) and 81% to 90%V_max_ speed band (*P* < 0.01, *r* = 0.58). However, for the 91% to 100%V_max_ speed band, the difference in running volumes between preseason and in-season was not significant despite a small effect size (*P* = 0.103, *r* = 0.22), likely owing to the small observed running volumes near maximal speed (Figure 1). The average weekly total distance and distance in absolute velocity bands are displayed in Table 3, with distance in relative velocity bands presented in Table 4.

**Table 3.** Pooled results of total distance and absolute speed bands (mean ± SD) during the preseason and in-season

			Distance Covered, m				
Weeks		Total Distance	<5.5 m s^-1^	5.51-7 m s^-1^	7.01-8.5 m s^-1^	8.51-10 m s^-1^	>10 m s^-1^
Preseason	1	33,539	30,196 (90%)^a^	2726 (8%)	623 (2%)	7 (<1%)	0 (<1%)
	2	40,289	36,105 (90%)	3478 (9%)	697 (2%)	8 (<1%)	0 (<1%)
	3	29,804	26,516 (89%)	2806 (9%)	479 (2%)	3 (<1%)	0 (<1%)
	4	28,098	25,114 (89%)	2547 (9%)	431 (2%)	7 (<1%)	0 (<1%)
	5	26,094	24,040 (92%)	1783 (7%)	263 (1%)	5 (<1%)	0 (<1%)
	6	34,747	32,488 (93%)	1931 (6%)	317 (1%)	11 (<1%)	0 (<1%)
	7	33,517	30,986 (92%)	2204 (7%)	318 (1%)	9 (<1%)	0 (<1%)
	8	29,836	27,718 (93%)	1786 (6%)	325 (1%)	7 (<1%)	0 (<1%)
	9	22,781	21,246 (93%)	1371 (6%)	159 (1%)	5 (<1%)	0 (<1%)
Mean ± SD		30,967 ± 5195	28,628 ± 4621 (92%)	2293 ± 646 (7%)	401 ± 174 (1%)	7 ± 2 (0%)	0 ± 0 (0%)
In-season	10	23,854	21,951 (92%)	1665 (7%)	234 (1%)	5 (<1%)	0 (<1%)
	11	34,929	32,811 (94%)	2560 (7%)	313 (1%)	14 (<1%)	0 (<1%)
	12	33,880	31,407 (93%)	2144 (6%)	318 (1%)	11 (<1%)	0 (<1%)
	13	29,348	27,210 (93%)	1838 (6%)	296 (1%)	4 (<1%)	0 (<1%)
	14	20,255	18,656 (92%)	1413 (7%)	183 (1%)	3 (<1%)	0 (<1%)
	15	33,094	31,018 (94%)	1823 (6%)	247 (1%)	5 (<1%)	0 (<1%)
	16	31,250	28,987 (93%)	1982 (6%)	272 (1%)	9 (<1%)	0 (<1%)
	17	32,667	30,676 (94%)	1776 (5%)	211 (1%)	5 (<1%)	0 (<1%)
Mean ± SD		29,909 ± 5219	27,839 ± 5019 (93%)	1900 ± 342 (6%)	259 ± 49 (1%)	7 ± 4 (0%)	0 ± 0 (0%)

a Percentage refers to the percentage of total volume.

**Table 4.** Pooled results of total distance and relative speed bands (mean **±** SD) during the preseason and in-season

Week		Total Distance, m	Distance Covered Above %V_max_, m									
			<70	71-75	76-80	81-85	86-90	91-95	96-100	71-80	81-90	91-100
Preseason	1	33,539	31,859 (95.0%)^a^	593 (1.8%)	318 (0.9%)	174 (0.5%)	578 (1.7%)	15 (0.0%)	1 (0.0%)	1059 (3.2%)	169 (0.5%)	17 (0.0%)
	2	40,289	38,232 (94.9%)	1349 (3.3%)	400 (1.0%)	192 (0.5%)	82 (0.2%)	30 (0.1%)	4 (0.0%)	2007 5.0%)	307 (0.8%)	34 (0.1%)
	3	29,804	28,688 (96.3%)	601 (2.0%)	316 (1.1%)	130 (0.4%)	49 (0.2%)	20 (0.1%)	0 (0.0%)	1033 (3.5%)	198 (0.7%)	20 (0.1%)
	4	28,098	27,138 (96.6%)	438 (1.6%)	240 (0.9%)	170 (0.6%)	92 (0.3%)	15 (0.1%)	5 (0.0%)	975 (3.5%)	303 (1.1%)	20 (0.1%)
	5	26,094	25,557 (97.9%)	253 (1.0%)	147 (0.6%)	82 (0.3%)	39 (0.1%)	14 (0.1%)	2 (0.0%)	510, 2.0%)	136 (0.5%)	16 (0.1%)
	6	34,747	33,880 (97.5%)	454 (1.3%)	252 (0.7%	104 (0.3%)	44 (0.1%)	13 (0.0%)	1 (0.0%)	802 (2.3%)	156 (0.4%)	14 (0.0%)
	7	33,517	32,715 (97.6%)	428 (1.3%)	220 (0.7%)	99 (0.3%)	38 (0.1%)	12 (0.0%)	5 (0.0%)	763 (2.3%)	150 (0.4%)	16 (0.0%)
	8	29,836	29,168 (97.8%)	338 (1.1%)	179 (0.6%)	100 (0.3%)	38 (0.1%)	10 (0.0%)	4 (0.0%)	617 (2.1%)	151 (0.5%)	14 (0.0%)
	9	22,781	22,310 (97.9%)	243 (1.1%)	122 (0.5%)	67 (0.3%)	26 (0.1%)	9 (0.0%)	2 (0.0%)	462 (2.0%)	104 (0.5%)	11 (0.1%)
Mean ± SD		30,967 ± 5195	29,946 ± 4784 (96.7%)	522 ± 335 (1.7%)	244 ± 90 (0.8%)	124 ± 45 (0.4%)	110 ± 177 (0.4%)	18 ± 6 (0.1%)	3 ± 2 (0.0%)	914 ± 464 (3.0%)	186 ± 72 (0.6%)	18 ± 7 (0.1%)
In-season	10	23,854	23,238 (97.4%)	320 (1.3%)	161 (0.7%)	79 (0.3%)	40 (0.2%)	15 (0.1%)	1 (0.0%)	583 (2.4%)	132 (0.6%)	16 (0.1%)
	11	34,929	34,243 (98.0%)	358 (1.0%)	180 (0.5%)	85 (0.2%)	43 (0.1%)	15 (0.0%)	4 (0.0%)	618 (1.8%)	143 (0.4%	19 (0.1%)
	12	33,880	33,091 (97.7%)	393 (1.2%)	216 (0.6%)	111 (0.3%)	45 (0.1%)	18 (0.1%)	4 (0.0%)	760 (2.2%)	177 (0.5%)	22 (0.1%)
	13	29,348	28,584 (97.4%)	362 (1.2%)	225 (0.8%)	121 (0.4%)	43 (0.1%)	11 (0.0%)	2 (0.0%)	705 (2.4%)	172 (0.6%)	13 (0.0%)
	14	20,255	19,843 (98.0%)	230 (1.1%)	115 (0.6%)	47 (0.2%)	17 (0.1%)	3 (0.0%)	0 (0.0%)	385 (1.9%)	66 (0.3%)	4 (0.0%)
	15	33,094	32,371 (97.8%)	379 (1.1%)	192(0.6%)	101 (0.3%)	35 (0.1%)	12 (0.0%)	4 (0.0%)	674 (2.0%)	148 (0.4%)	16 (0.0%)
	16	31,250	30,298 (97.0%)	556 (1.8%)	231 (0.7%)	107 (0.3%)	45 (0.1%)	9 (0.0%)	3 (0.0%)	874 (2.8%)	159 (0.5%)	12 (0.0%)
	17	32,667	32,011 (98.0%)	348 (1.1%)	175 (0.5%)	89 (0.3%)	32 (0.1%)	10 (0.0%)	2 (0.0%)	605 (1.9%)	130 (0.4%)	13 (0.0%)
Mean ± SD		29,909 ± 5219	29,209 ± 5116 (97.7%)	368 ± 91 (1.2%)	186 ± 38 (0.6%)	93 ± 23 (0.3%)	38 ± 114 (0.1%)	12 ± 5 (0.0%)	3 ± 2 (0.0%)	650 ± 143 (2.2%)	141 ± 35 (0.5%)	14 ± 5 (0.0%)

a Percentage refers to the percentage of total volume.

The average weekly running volume for Club A was significantly different between the preseason and in-season periods at the 71% to 80%V_max_ (*P* < 0.01, *r* = 0.59), 81% to 90%V_max_ (*P* < 0.01, *r* = 0.77), and 91% to 100%V_max_ (*P* = 0.03, *r* = 0.44). However, for Club B, the average weekly running volume was significantly different between the preseason period and the in-season period only within the speed band 71% to 80%V_max_ (*P* = 0.01, *r* = 0.55). For speed bands 81% to 90%V_max_ (*P* = 0.19, *r* = 0.28) and 91% to 100%V_max_ (*P* = 0.28, *r* = 0.21), the differences were not statistically significant.

**Figure 2.**
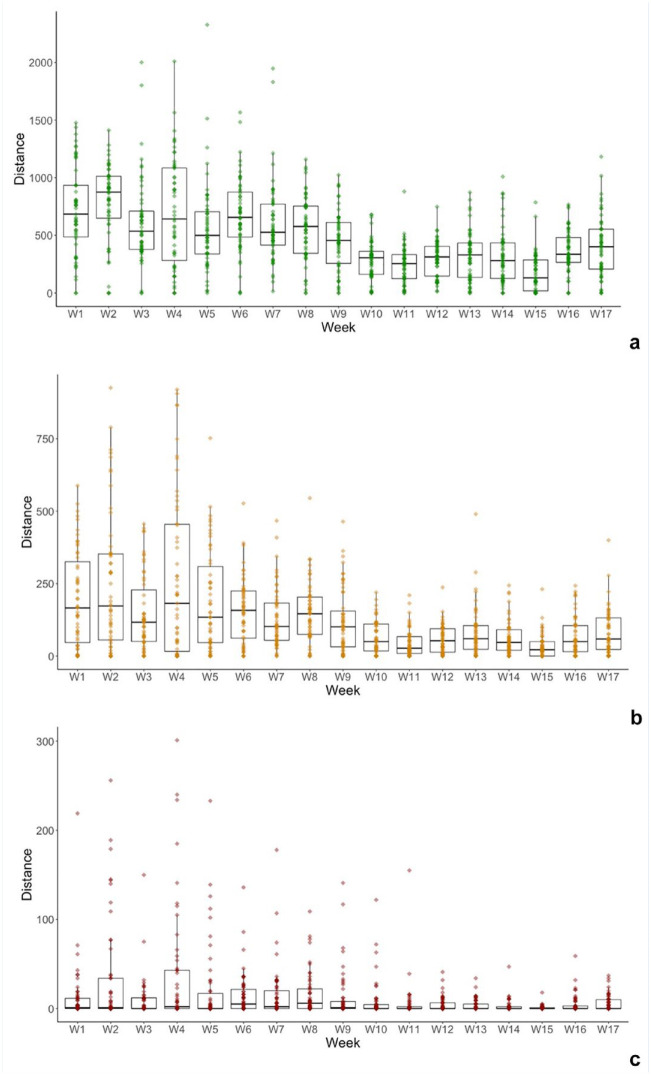
Weekly distance in the (a) 71% to 80%V_max_, (b) 81% to 90%V_max_, and (c) 91% to 100%V_max_ speed bands presented for the preseason (weeks 1 to 9) and the initial 8 weeks of the season (weeks 10 to 17). Individual dots represent each athlete during each week.

**Figure 3.**
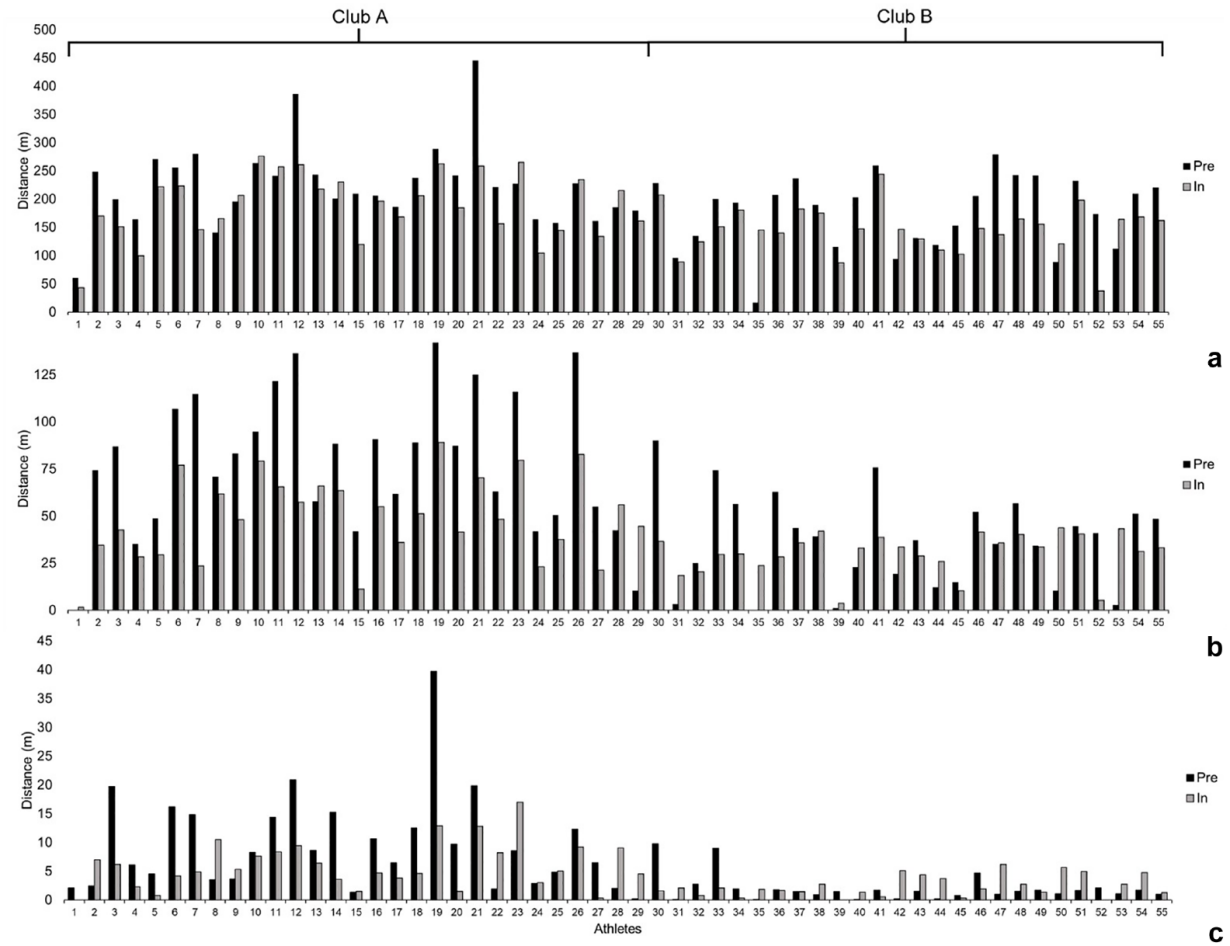
Average weekly distance between (a) 71% to 80%V_max_, (b) 81% to 90%V_max_, and (c) 91% to 100%V_max_ for all athletes across the preseason and first 8 rounds of the 2019 season. Club A is represented by P1-29, whereas Club B is P30-55.

Differences in weekly distance covered at 71% to 80%V_max_, 81% to 90%V_max_, and 91% to 100%V_max_ are shown in Figure 2. Individual variation is shown for all athletes at speeds >80%V_max_ and then speeds >90%V_max_ (Figure 3). Of the 55 athletes in this study, 41 (75%) completed more distance >80%V_max_ in the preseason compared with the in-season. Similarly, 33 (60%) athletes completed more distance >90%V_max_ in the preseason when compared with the in-season. To elucidate the individual variation of athletes from HSR thresholds, the coefficient of variation (CV) was calculated for the 71% to 80%V_max_ (CV, 51%), 81% to 90%V_max_ (CV, 39%), and 91% to 100%V_max_ (CV, 41%) thresholds. A key finding is that 6 athletes did not achieve >95%V_max_ in the preseason, whereas 7 different athletes did not achieve >95%V_max_ during the first 8 rounds of the season.

## Discussion

This study investigated the amount of running completed by professional AF athletes in relative speed bands during the preseason and first 8 rounds of the competitive season. In addition to this, individual variation for athletes was reported for both absolute and relative speed bands. These findings reveal that professional AF athletes completed much of their running at speeds below 70%V_max_. On a broad level, the total volume of HSR in the preseason appears to match the competitive demands of the in-season. This statement should be qualified in context, as there is no current evidence of the appropriate dose to adequately elicit performance gains and mitigate injury risk in this population. A potential explanation for this is the large number of variables that influence training dose in a field sport such as AF.

Closer examination highlights that the volume of HSR is much greater in the initial 4 weeks of the preseason than during any other point in the preseason or competitive phases. Although this finding appears to be counterintuitive from a progressive overload standpoint, it may be rationalized by an understanding of the constraints to training availability during the Christmas period (the main off-season period in Australia) and the time immediately succeeding it. Similarly, the amount of sprint running peaked in week 2 of the preseason before decreasing gradually until the season began. Individual variation was high for the population sample in the study; coefficients of variation exceeded 30% in all cases,^48^ with a weekly average ranging from 0 m to 180 m above 90%V_max_ in the preseason (Figure 2). Generally, the findings from this investigation suggest that preseason running volume completed by athletes in this study adequately matches the volume of the in-season period. However, the variance in volumes suggests that some athletes may be underprepared for the demands of competition. It is unknown if this volume mediates the injury risk, but it most likely represents an insufficient stimulus to elicit a training response.

**Figure 4.**
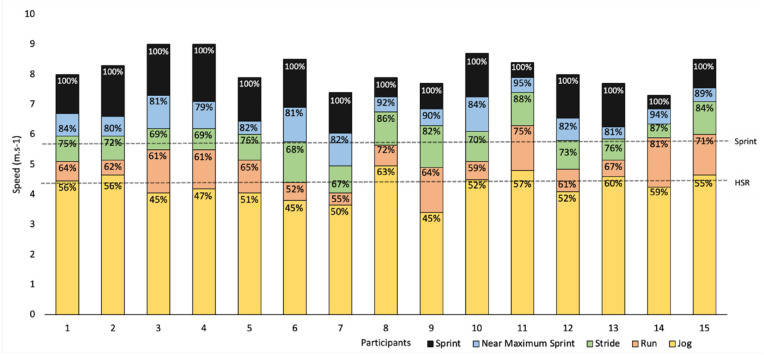
The participant range of absolute and relative speeds in comparison with a standardized set of absolute HSR and sprinting threshold. This graph highlights that it is possible to achieve the HSR threshold using a jogging gait, and the sprinting threshold when completing a stride. The arbitrary HSR and sprint lines are derived from Table 2. HSR, high-speed running.

The athletes involved in this study completed an average weekly volume of 30,967 m ± 5195 m during the preseason period of the 2019 AFL season (Table 3). Of this volume, 96.7% was completed at speeds <70%V_max_. Furthermore, a large amount is completed by walking, indicating that total volume is a poor metric for conditioning prescription. This is consistent with other work indicating that professional AF is dictated by long periods of low intensity movement interspersed with short high intensity bursts.^26^ Analysis of relative speed bands indicated that the average weekly volume during preseason was 914 m above 70%V_max_, 186 m above 80%V_max_, and 18 m above 90%V_max_. Findings from previous investigations indicate that AFL teams regularly use 80%V_max_ as threshold for sprint running.^20^ Recently, Freeman et al^19^ highlighted both the individual variation of sprinting and the similarities between 80%V_max_ and the sprinting threshold (Figure 4). This should be considered in conjunction with evidence from analyses that suggest that there is a large nonlinear increase in biomechanical variables between 80%V_max_ and 100%_Vmax_; therefore, 80%V_max_ is most likely not high-speed from a lower-limb musculature perspective.^8,16,38,43^

In terms of total volume, athletes travelled similar weekly distances in preseason (30,967 m) and the in-season (29,909 m). However, the composition of this running volume changed slightly with total distance completed between 70% to 80%V_max_, 80% to 90%V_max_, and 90% to 100%V_max_ all significantly (*P* < 0.05) lower in-season. A shift in focus of training goals may be partly responsible for the drop in overall volume, particularly in the slower HSR running thresholds. This is because greater emphasis is placed upon recovery from fatigue associated with the increased intensity of competition and maintenance of physical qualities to enable optimum performance.^28^. Another contributing factor in the reduction in overall volume is the increased variability of match demands, where factors such as positional differences, tactical decisions, and score line influence the physical profile of each individual match.^21,35,41^ These findings partially support the primary hypothesis in which athletes would complete significantly less HSR and sprinting volume during the in-season phase when compared with the preseason.

Analyses of both Club A and Club B challenge the pooled findings, specifically as there are no significant reductions in volume above 90%V_max_ for Club A and 80%V_max_ for Club B. Broadly, this could be explained by the difference in training exposure between Club A and B (Table 1) but also the different periodization approaches of the physical performance staff delivering the sessions. High-performance managers in the AFL have previously acknowledged that they reduce the dedicated sprinting exposure in-season to accommodate for the other demands (eg, contact) of the match.^20^ The findings from this study would suggest that this is true of slower thresholds but not of sprinting (defined as >90%V_max_ in this study).

Previous investigations indicate that AFL teams are using thresholds as low as 80%V_max_ to record sprint distance.^20^ Figures 1, 2, and 3 display the large variation for individual athletes across the range of 71% to 80%V_max_, 81% to 90%V_max_, and 91% to 100%V_max_. During preseason, 5 (11%) athletes exceeded the average 91% to 100%V_max_ by >10m per week. Conversely, 8 (15%) athletes achieved ≤2m per week in the 91% to 100%V_max_ during the preseason period. This would indicate that the broad analysis of the means suggests that the overall cohort of athletes was adequately prepared during the preseason to perform during the initial 8 rounds of competition. This closer analysis shows that some athletes are “overachieving” and inflating the group average (Figure 3). Similarly, several athletes were not regularly exposed to any sprinting in the 91% to 100%V_max_ threshold (Figure 3c). As sprinting at maximum speed does occur in AF, this suggests that many athletes may have been underprepared during the preseason. This means that performance may be inhibited and the risk of soft-tissue injury may be increased.

During the in-season phase, 30 athletes completed more distance in the 91% to 100%V_max_ threshold than in the preseason. A plausible explanation is that the commencement of competition increased the effort of athletes—a theory previously reported in existing literature.^2^ However, the pooled distance for both clubs decreased for the >91%V_max_ threshold. This indicates that overall volumes were low in both periods, and overall changes are influenced heavily by individual athletes in a small sample size. Perhaps another interesting finding is the discrepancy in maximum speed between training and matches. To complement this, 36 athletes in this study recorded a faster V_max_ during the first 8 rounds of the season than the preseason. This might be because athletes that obtained consistent HSR and sprint exposure in preseason got faster as the season progressed. Perhaps this is explained again by the competitive effect^24^ or because coaches are relying on match-play as the exposure to maximum speed in-season.^2^ However, this poses a potentially dangerous situation. If the athlete only achieves higher speeds in a match, this may place the athlete at an increased risk of injury, especially if they have not previously reached those speeds consistently before competition.

## Practical Implications

In a broad sense, the key practical application from this study is that HSR and sprinting exposure are highly variable across professional AF. More consistent exposure to sprinting has 2 proposed benefits: an increased maximum speed and potential subsequent performance benefit, a prophylactic effect for soft-tissue injury, as it likely acts to condition the lower-limb muscles for the increased mechanical demands.^8,16,31,38,43^ Similarly, the existing technology allows sport science staff at an elite level to identify athletes who are heavily underexposed to maximum speed running. These athletes would likely attain some benefits, both in performance and injury prevention, by increasing their maximum speed sprint exposure closer to the team average. The authors acknowledge, however, that several different factors influence the ability of an athlete to run fast consistently in a high-contact sport such as professional AF. Therefore, adherence to resistance training exercises with a strong evidence base remains recommended in this population. As in most instances, further investigation is warranted into the sprinting and HSR demands of elite AF clubs. A prospective longitudinal study that investigates detailed training load and tracks injury would be an insightful resource for practitioners working in AF but also other similar field-sports with contact and an HSR component. In addition, further information identifying positional differences and how the different volumes are accrued, ie, training drills, small-sided games, or match play, would further allow practitioners and academics to investigate the multifactorial aspects of performance and injury. Finally, a retrospective analysis that investigates injured athletes and describes their HSR and sprinting exposure through the use of GPS would also be of value to practitioners.

## Supplemental Material

sj-docx-1-sph-10.1177_19417381241265114 – Supplemental material for Preseason and In-Season High-Speed Running Demands of 2 Professional Australian Rules Football Teams Supplemental material, sj-docx-1-sph-10.1177_19417381241265114 for Preseason and In-Season High-Speed Running Demands of 2 Professional Australian Rules Football Teams by Brock W. Freeman, Scott W. Talpey, Lachlan P. James, Russell J. Rayner and Warren B. Young in Sports Health
