# Supplementary material for: Preseason and In-Season High-Speed Running Demands of 2 Professional Australian Rules Football Teams
Source: Sports Health. 2024 Aug 22;17(1):27–38. doi: 10.1177/19417381241265114 (PMC11799764; doi:10.1177/19417381241265114)
Supplement: sj-docx-1-sph-10.1177_19417381241265114 – Supplemental material for Preseason and In-Season High-Speed Running Demands of 2 Professional Australian Rules Football Teams [file sj-docx-1-sph-10.1177_19417381241265114.docx]

**Online Appendix**


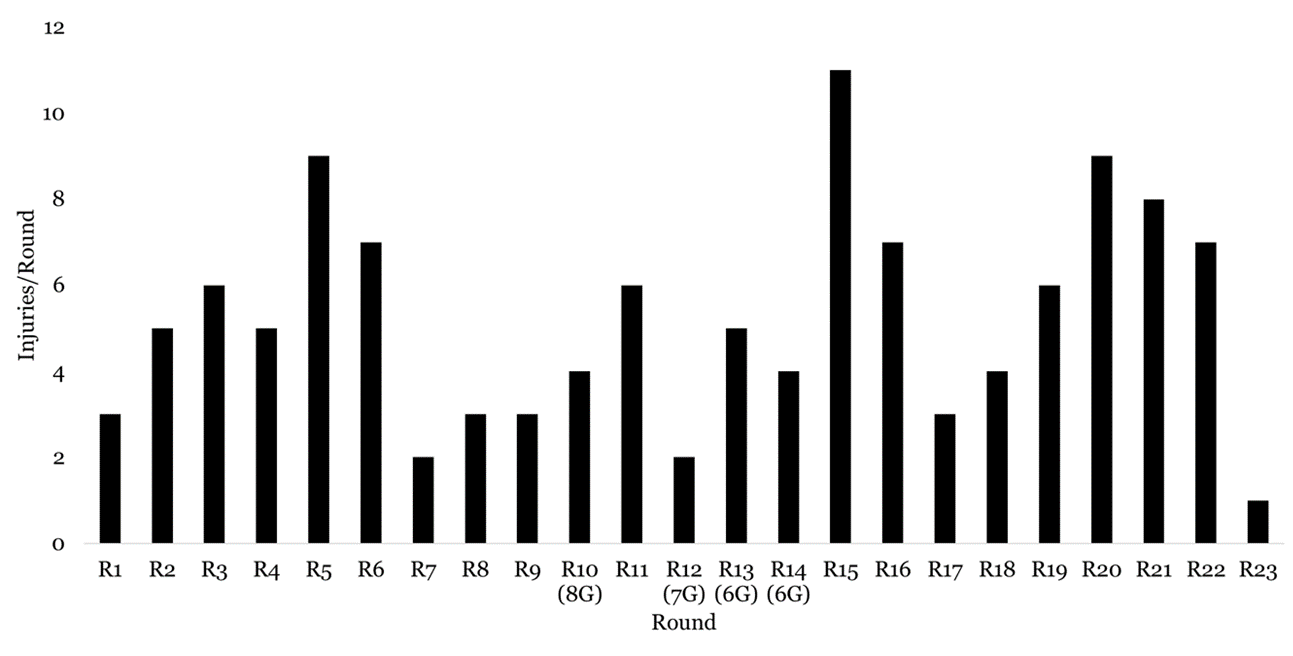


**Figure A1.** Hamstring strain injuries for each round of the 2018 AFL season. The letter G reflects the number of games in the respective round. A large increase is observed in the initial third of the season. Source: Publicly available data collected from the AFL’s publicly available Weekly Injury Report. Retrieved from https://www.afl.com.au/matches/injury-list - 22/1/2018 through 30/9/2018.
